# Transferrin as a thermosensitizer in radiofrequency hyperthermia for cancer treatment

**DOI:** 10.1038/s41598-018-31232-9

**Published:** 2018-09-10

**Authors:** Hea-Jong Chung, Heui-Kwan Lee, Ki Beom Kwon, Hyeon-Jin Kim, Seong-Tshool Hong

**Affiliations:** 1 Department of Biomedical Sciences and Institute for Medical Science, Chonbuk National University Medical School, Jeonju, Chonbuk 54907 South Korea; 2 Deparment of Radiation oncology, Presbyterian Medical Center, Seonam University Medical School, Jeonju, Chonbuk 54987 South Korea; 3 School of Public Health, University of Washington, Seattle, Washington United States of America; 4 JINIS BDRD institute, JINIS Biopharmaceuticals Co., 913 Gwahak-Ro, Bongdong, Wanju, Chonbuk 55321 South Korea

## Abstract

One of the main characteristics of cancer tissues is poor development of neovascularization that results in a limited blood circulation. Because of this phenomenon, it is harder for cancer tissues to diffuse their elevated heat into other parts of the body. The scientific principle of radiofrequency hyperthermia relies on this quality of cancer tissues which with higher temperature becomes more apparent. Despite the obvious necessity to selectively heat the cancer tissue for radiofrequency hyperthermia, a proper thermosensitizer has not been developed until now. Here, we show that transferrin containing ferric ion could be an ideal thermosensitizer for the increased efficiency of radiofrequency hyperthermia. In our result, the ferric ion-enriched cancer tissues dramatically react with 13.56 MHz radiofrequency wave to cause cancer-selective dielectric temperature increment. The overall anticancer efficacy of a 13.56 MHz radiofrequency hyperthermia using transferrin as a thermosensitizer was much higher than the oncotherapeutic efficacy of paclitaxel, successfully eradicating cancer in a tumor-xenografted mouse experiment.

## Introduction

The uncontrolled, rapid growth of cancer cells not only leads to formation of an unorganized cell mass but also makes it impossible to develop functional neovascularization as in the case of normal tissues^1,2^. Thus, cancer cells suffer from an inadequate blood supply, resulting in chronic hypoxia which has been frequently observed in cancer tissues indicated by lowered pH due to increased glycolysis and poor diffusion of nutrient and chemicals such as chemotherapeutic agents. Improper development of vascularization in cancer tissues also limits blood transportation, leading to cancer tissues’ impaired ability to diffuse heat. While elevated heat in normal tissue can rapidly diffuse into the other part of body through the circulatory system, poor vascularization in cancer tissues causes heat to be accumulated within the region, making the cancer tissue vulnerable to heat elevation^3^. Therefore, hyperthermal cancer treatments were designed based on such morphological and physiological characteristics of cancer tissues^4^.

Among the various treatments relying on the elevation of temperatures in cancer tissues, radiofrequency hyperthermia has been the most widely adopted in current cancer therapies^5^. However, despite its widespread application in modern cancer therapies, it is not possible to selectively elevate the temperature of cancer cells by current radiofrequency hyperthermia. Because of this drawback, current radiofrequency hyperthermia aims to elevate the temperature of cancer tissues just to the range of 40~42 °C so that the surrounding normal tissues do not get damaged^6^. However, such a small elevation in temperature is not enough to kill cancer cells, and the oncotherapeutic efficacy of current radiofrequency hyperthermia is minor in cancer treatment^7,8^.

Radiofrequency is a kind of electromagnetic wave with frequency that lies in the range from around 3 kHz to 300 GHz. Electromagnetic waves are already widely used in modern cancer therapies. X-ray and red light are the best examples^9,10^. However, both X-ray and red light are not effective in treating cancer if they are used without their corresponding sensitizing reagents, *i*.*e*. radiosensitizers for X-ray and photosensitizers for red light^11,12^. Although radiofrequency hyperthermia is solely used without a sensitizing agent in current cancer therapies, it is gaining popularity in current cancer treatments because of its moderate oncotherapeutic efficacies^13–15^. Considering the fact that electromagnetic waves such as X-ray and red light require sensitizing agents for efficient cancer treatment, it would be reasonable to speculate that radiofrequency hyperthermia combined with a thermosensitizer of their own could be much more effective in cancer treatment.

Metal ions were observed to interact well with radiofrequency waves to generate strong dielectric heat^16,17^. Ferric ion, which has a very strong dipole moment, induces especially strong dielectric heating in radiofrequency waves^18^. Transferrin, a ferric ion-binding blood plasma glycoprotein, is able to target cancer cells^19^, which means that transferrin could be used to deliver ferric ions to cancer tissues. Theoretically, accumulated ferric ion in cancer cells would allow radiofrequency waves to generate concentrated dielectric heat for treatment of cancer. Considering these characteristics of transferrin and ferric ion, we investigated a possibility of using transferrin as a thermosensitizer for a radiofrequency hyperthermia. Our experimental data show that transferrin is able to selectively deliver ferric ion into cancer cells, and animal experiments reveal that using transferrin as a thermosensitizer in a radiofrequency hyperthermia eradicated cancer completely.

## Results

### Transferrin, a kind of glycoprotein containing ferric ion, was able to successfully react with radiofrequency waves to generate strong dielectric heat

**Figure 1:**
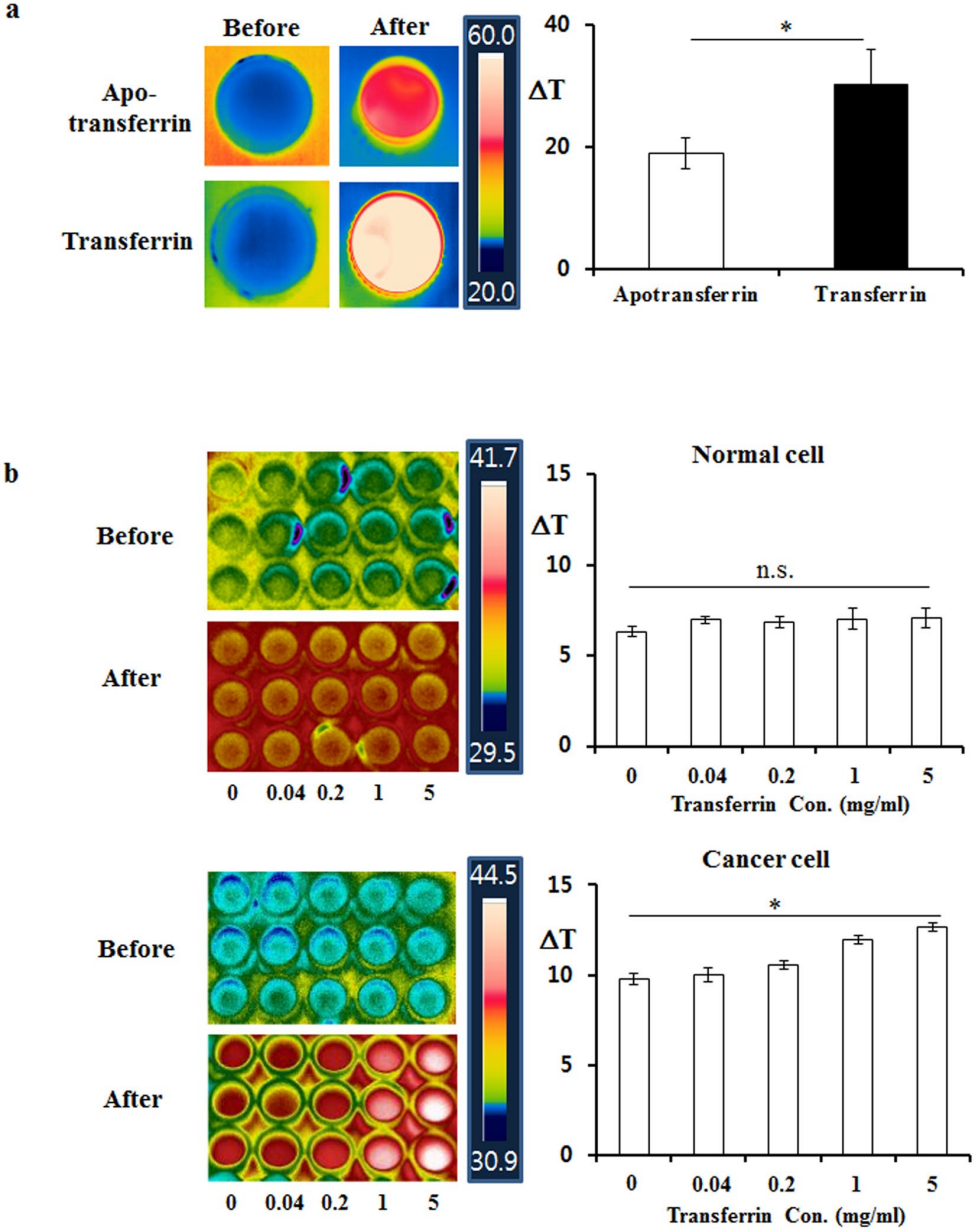
Ferric ion-containing transferrin induced dielectric heat in the 13.56 MHz radiofrequency wave to boost temperature elevation *in vitro*. (**a**) Either apotransferrin or transferrin solutions of the indicated amount were irradiated by the same amount of 13.56 MHz radiofrequency wave under the same condition, and temperature changes were indicated. The temperature was recorded by FLIR thermal camera before and after 13.56 MHz radiofrequency hyperthermia and temperature change (ΔT) was calculated. The transferrin solution containing ferric ion showed significant temperature elevation compared to apotransferrin solution without ferric ion. (**b**) Exposure of human lung cancer NCI-H460 cells to transferrin boosted temperature elevation in a dose dependent manner while this effect was not observed in normal primary cells. The values represent the mean ± standard deviation (*n* = 6). A paired Student’s t-test was used for the statistical analysis; *p < 0.05.

Ferric ion has a very strong dipole moment, allowing it to induce strong dielectric heat when heated with radiofrequency waves in theory. To confirm this, we performed a heat induction experiment on transferrin solution using radiofrequency wave. As expected, the temperature of the transferrin solution increased more than the temperature of the solution containing apotransferrin without ferric ion did when irradiated with the same amount of the 13.56 MHz radiofrequency wave (Fig. 1a). This *in vitro* result confirmed that the ferric ion in transferrin is able to generate dielectric heat.

It has been well known that transferrin has a strong cancer-targeting ability^16,19^, which means that in the presence of transferrin, concentration of ferric ion should be higher in cancer tissues than in normal tissues. Thus, if both types of cells were exposed to a transferrin solution beforehand, heating with radiofrequency waves would induce more dielectric heat in tumor cells than in normal cells. Figure 1b shows that a brief exposure of cancer cells to a medium containing transferrin boosted temperature elevation in a dose-dependent manner in contrast to normal primary cells (see Supplementary Fig. 1). Overall, these experiments indicate that ferric ion, selectively accumulated in cancer cells, reacted with the radiofrequency wave to induce dielectric heat more efficiently, suggesting that transferrin would be an ideal choice for radiofrequency hyperthermia as a thermosensitizer.

### Injection of transferrin into mice bearing tumor successfully resulted in selective accumulation of ferric ion in cancer cells

**Figure 2:**
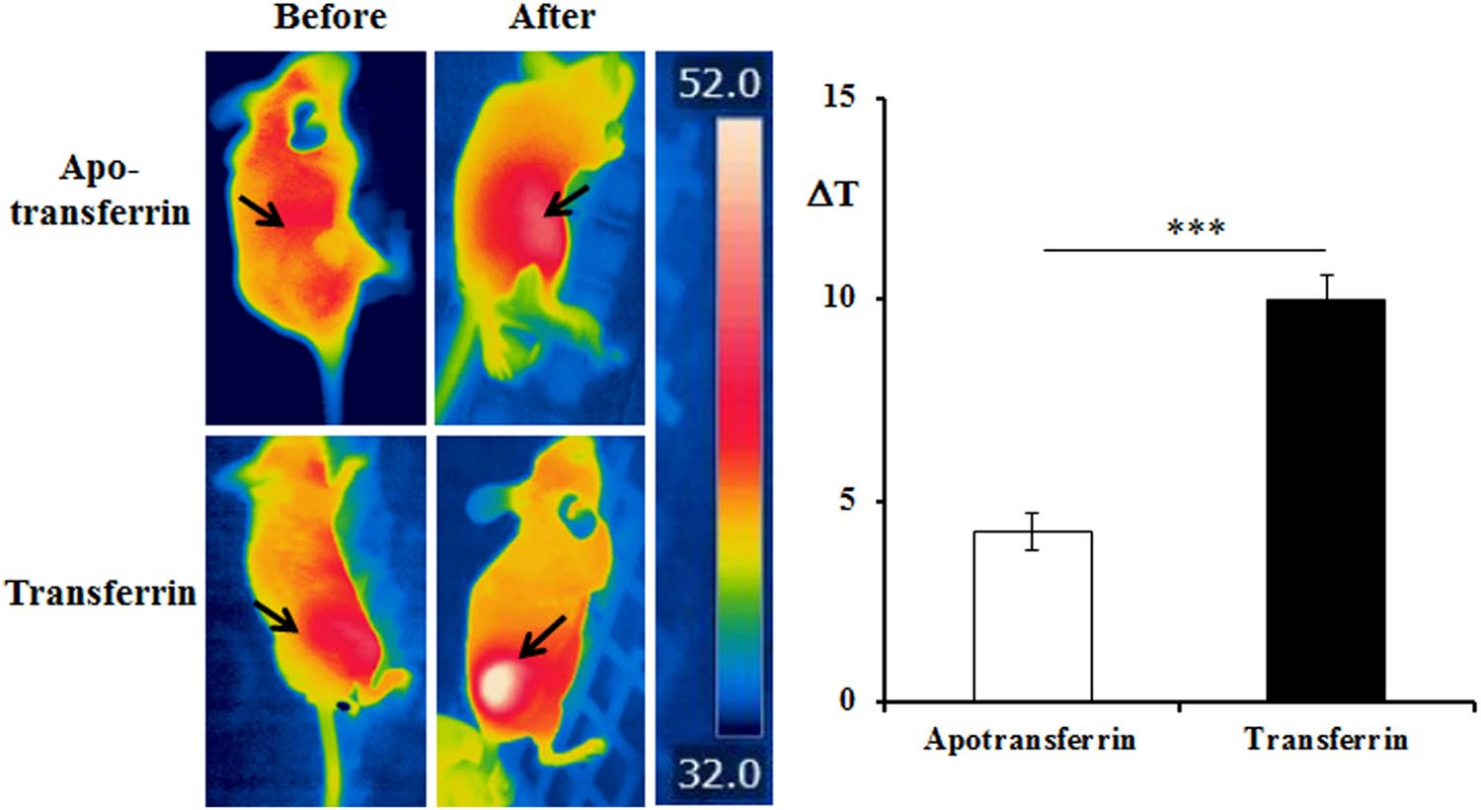
Ferric ion-containing transferrin boosted temperature elevation in tumor-bearing mice in the 13.56 MHz radiofrequency hyperthermia *in vivo*. Tumor-bearing mice were given *i*.*v*. injection with either apotransferrin or transferrin and the temperature of tumor tissues was recorded before and after 13.56 MHz radiofrequency hyperthermia for temperature change (ΔT) calculation. The temperature elevation in tumor tissue was dramatically boosted by local hyperthermia using a 13.56 MHz radiofrequency hyperthermia with transferrin but not with apotransferrin. The values represent the mean ± standard deviation (*n* = 6). A paired Student’s t-test was used for the statistical analysis; ***p < 0.001.

**Figure 3:**
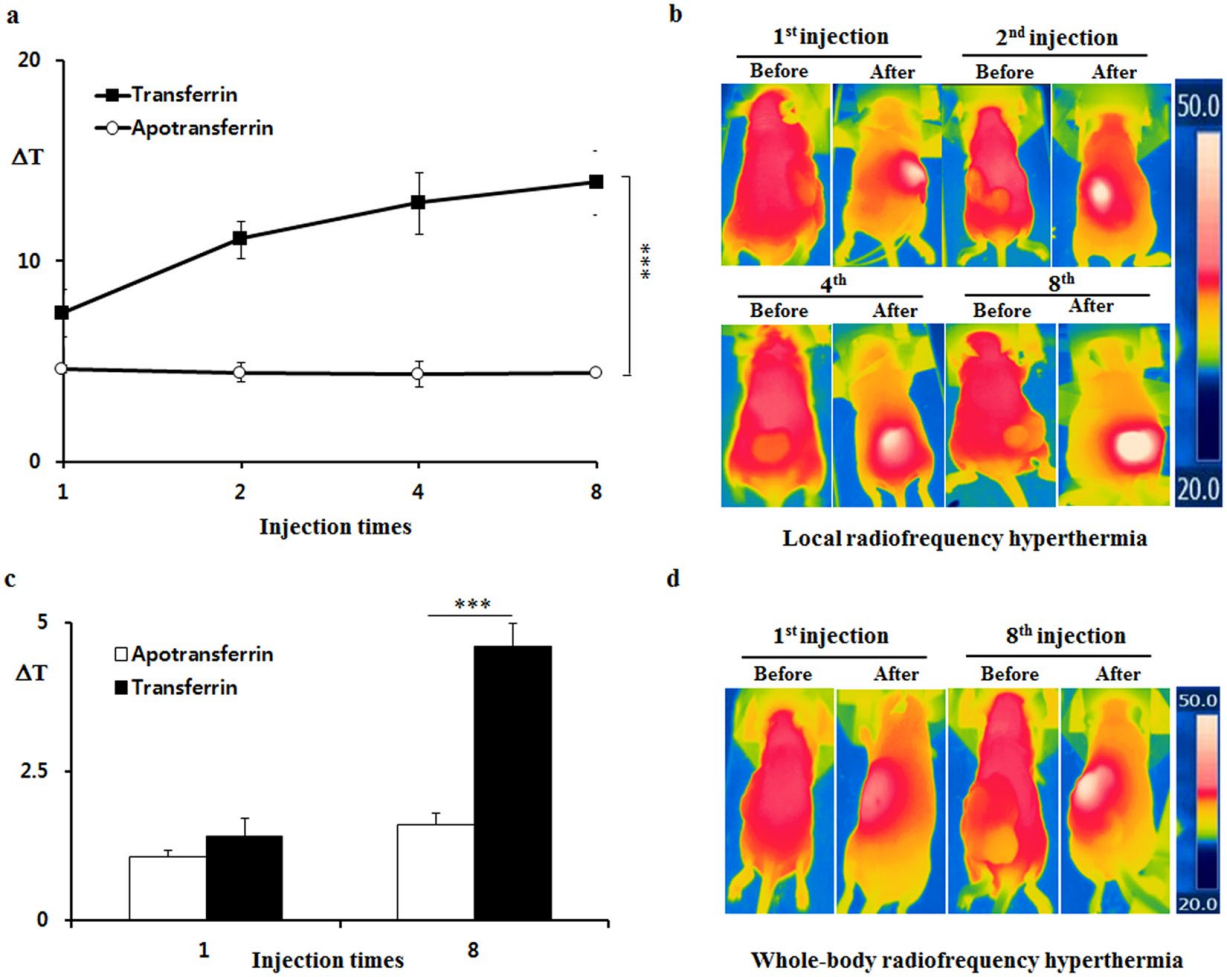
The cancer tissues became more reactive to 13.56 MHz radiofrequency hyperthermia as *i*.*v*. injections of transferrin were repeated. (**a**) The repeated *i*.*v*. injections of transferrin to tumor-bearing mice boosted temperature elevation in a local 13.56 MHz radiofrequency hyperthermia as ferric ion accumulated (see Supplementary Fig. 2). (**b**) The representative images of (a). (**c**) The repeated *i*.*v*. injection of transferrin boosted temperature elevation in a whole-body 13.56 MHz radiofrequency hyperthermia as ferric ion accumulation increased. (**d**) The representative images of (c). The temperature was measured before and after 13.56 MHz radiofrequency hyperthermia for temperature change (ΔT) calculation. The values represent the mean ± standard deviation (*n* = 6). A paired Student’s t-test was used for the statistical analysis; ***p < 0.001.

**Figure 4:**
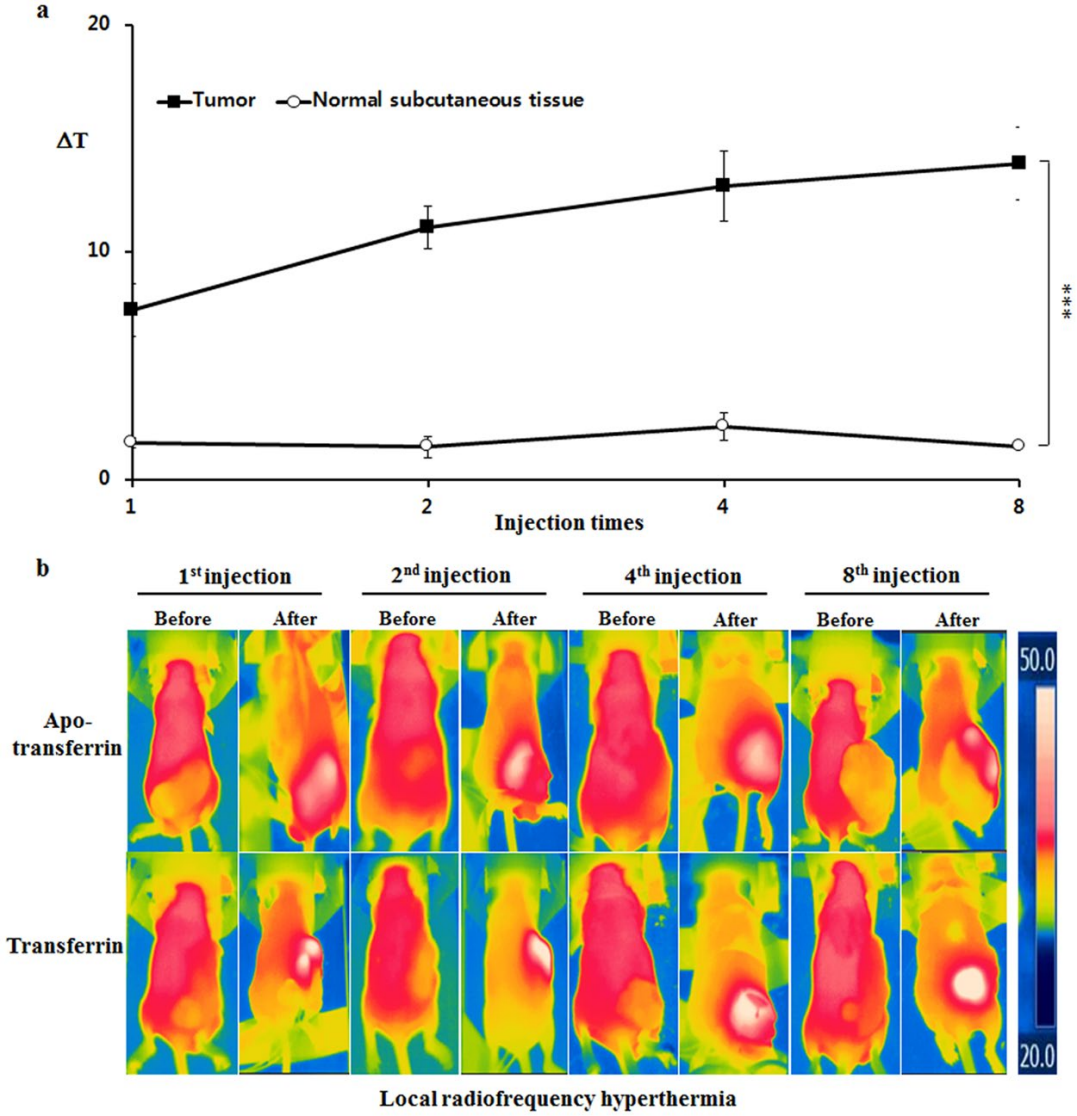
The selective temperature elevations of cancer tissue by a local 13.56 MHz radiofrequency hyperthermia was positively correlated with ferric ion accumulation. Accumulation of ferric ion in cancer cells was achieved by repeated *i*.*v*. injection of transferrin as described in Supplementary Fig. 2. The temperature of tumor tissues or normal subcutaneous tissues was measured before and after local hyperthermia for temperature change (ΔT) calculation. (**a**) The comparative analysis showed that accumulation of ferric ion by repeated *i*.*v*. injections of transferrin elevated temperature higher in cancer tissue than in normal subcutaneous tissues in a local hyperthermia. (**b**) Representative images of transferrin injection group were shown along with apotransferrin injection group. The values represent the mean ± standard deviation (*n* = 6). A paired Student’s t-test was used for the statistical analysis; ***p < 0.001.

**Figure 5:**
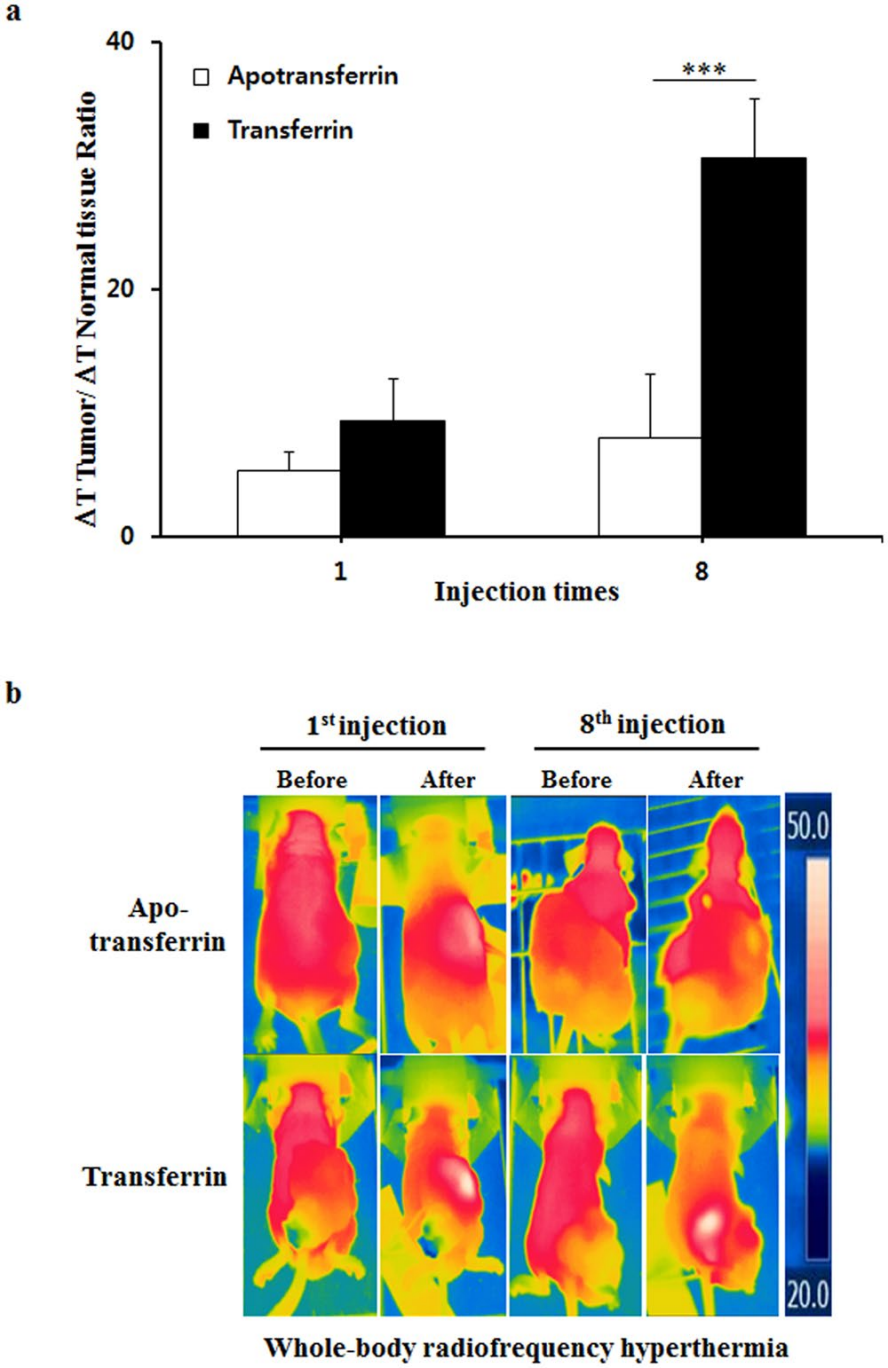
The selective temperature elevation of cancer tissue by a whole-body 13.56 MHz radiofrequency hyperthermia was positively correlated with ferric ion accumulation by repeated injections of transferrin. Accumulation of ferric ion in cancer cells was achieved by repeated *i*.*v*. injections of transferrin as described in Supplementary Fig. 2. The temperatures of tumor tissues in either apotransferrin or transferrin injection groups were measured before and after whole-body hyperthermia for temperature changes and relative ratio calculation. (**a**) The comparative analysis showed that ferric ion accumulation achieved by repeated *i*.*v*. injections of transferrin elevated temperature higher in cancer tissue compared to injections of apotransferrin in a whole-body hyperthermia. (**b**) Representative images of a. The values represent the mean ± standard deviation (*n* = 6). A paired Student’s t-test was used for the statistical analysis; ***p < 0.001.

The *in vitro* cell experiments in Fig. 1 show that the cancer-targeting characteristic of transferrin makes it a potential ferric ion-delivery vehicle for cancer-selective temperature elevation in radiofrequency hyperthermia. Transferrin receptors are known to be overexpressed in cancer cells^20–23^. However, it has never been reported whether injecting transferrin into cancer-bearing hosts causes cancer cells to increase transferrin uptake. Table 1 shows ferric ion distribution in cancer-bearing mice before and after *i*.*v*. injection of transferrin and apotransferrin, respectively. As expected from the fact that cancer cells overexpress transferrin receptor^20–23^, ferric ion selectively accumulated in cancer tissues after *i*.*v*. injection of transferrin (Table 1). *I*.*v*. injection of transferrin led to accumulation of ferric ion in the heart and liver also although ferric ion in the organs was not accumulated as much as in the tumor tissue. One of characteristics of cancer tissues is poor developments of vascularization which limits blood transportation. Although the innate limited blood transportation leads to cancer tissues’ impaired ability to diffuse heat and be accumulated within the cancer tissue to be vulnerable to heat elevation, elevated heat in normal tissue can rapidly diffuse into the other part of body through the circulatory system^3^. In fact, transferrin did not affect the temperature elevations in the heart and liver in radiofrequency hyperthermia while boosted dramatically temperature elevations in the cancer tissue (Figs 2–5 and see Supplementary Figs 6–9). As expected, repeated *i*.*v*. injection of transferrin into cancer-bearing mice resulted in a gradual increment of ferric ion concentration in cancer cells in a dose dependent manner (see Supplementary Fig. 2). These results clearly show that *i*.*v*. injection of transferrin into cancer-bearing mice leads to selective accumulation of ferric ion in cancer tissues.

**Table 1:** Distribution of ferric ion in each organ after single *i*.*v*. injection of transferrin into tumor-bearing mice.

	Apotransferrin (μg/g)	Transferrin (μg/g)	Fold increase
Tumor	13.5 ± 1.34	43.9 ± 9.7	**3**.**25**
Liver	74.5 ± 7.5	94.7 ± 8.1	1.27
Lung	28.3 ± 6.1	43.9 ± 17.9	1.55
Kidney	56.9 ± 7.5	41.2 ± 1.7	0.72
Heart	32.2 ± 1.8	70.1 ± 12.3	2.18
Muscle	29.5 ± 4.2	25.7 ± 5.2	0.87
Stomach	27.8 ± 3.8	46.4 ± 14.9	1.67
Brain	22.5 ± 3.7	28.4 ± 3.5	1.26

The ferric ion in each organ of the tumor-bearing mice was quantitated by the ICP-MS method after complete acid lysis of the organs. The values represent the mean ± standard deviation (*n* = 6).

### After injection of transferrin, the ferric ion-enriched cancer tissues dramatically react with 13.56 MHz radiofrequency wave to cause cancer-selective dielectric temperature increment

After confirming that ferric ion selectively accumulated in cancer cells using transferrin injection, we conducted experiments on cancer-bearing mice to confirm whether ferric ion would boost temperature elevation with radiofrequency hyperthermia. Two groups of cancer-bearing mice were used: one treated with *i*. *v*. injection of transferrin and the other treated with apotransferrin. A local 13.56 MHz radiofrequency hyperthermia after *i*.*v*. injection of transferrin increased the temperature of the cancer tissue of the mice by 10.3 °C, while the temperature only increased by 4.3 °C for the apotransferrin-treated group (Fig. 2 and see Supplementary Figs 3 and 6).

Since *in vitro* experiment showed a positive correlation between the ferric ion concentration and temperature elevation under the radiofrequency hyperthermia, the *in vivo* effect of accumulated ferric ion under the same condition was further investigated. Figures 3–5 show that cancer tissues reacted greater in 13.56 MHz radiofrequency hyperthermia as the concentration of ferric ion in cancer cells increased by repeated transferrin injections into the tumor-bearing mice. The local 13.56 MHz radiofrequency hyperthermia on the cancer-bearing mice after 8^th^ injection of transferrin resulted in a temperature elevation of 13.9 °C in the cancer tissue, while the temperature of apotransferrin-treated group’s cancer tissue only increased by 4.4 °C (Fig. 3a,b and see Supplementary Fig. 7). Similarly, a 13.56 MHz whole-body radiofrequency hyperthermia performed in the same condition increased the temperature of cancer tissue by 4.6 °C, while only 1.6 °C increased in the cancer tissue of the control group (Fig. 3c,d and see Supplementary Fig. 7). The relationship between intensity of heating and concentration of ferric ion became more evident when normal subcutaneous was compared to tumor tissue (Fig. 4 and see Supplementary Fig. 8). Temperature change has increased over repeated injection of transferrin in the tumor tissue but did not in normal subcutaneous tissues onto local radiofrequency hyperthermia (Fig. 4 and see Supplementary Figs 3, 4 and 8). In accordance to local hyperthermia, whole body hyperthermia after repeated injection of transferrin also resulted tumor-selective temperature elevation (Fig. 5 and see Supplementary Figs 5 and 9). These results clearly indicate that the selective accumulation of ferric ion in the cancer cells induced by *i*.*v*. injection of transferrin dramatically elevates intensity of dielectric heating in cancer cells in radio-frequency hyperthermia.

### In a tumor-xenografted mouse experiment to test on the oncotherapeutic efficacy of transferrin as a thermosensitizer, local radiofrequency hyperthermia combined with transferrin completely eradicated cancer

**Figure 6:**
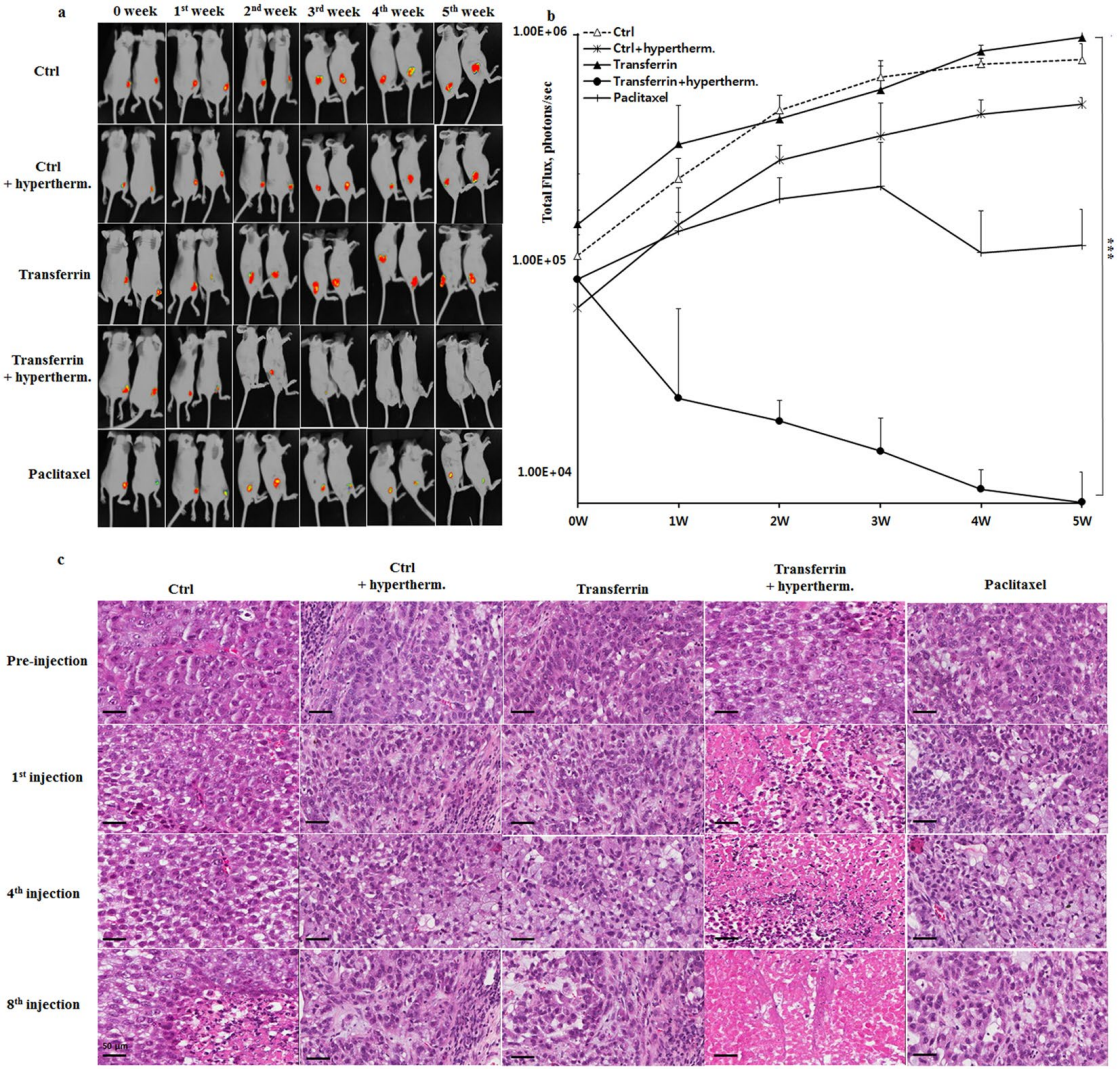
The oncotherapeutic efficacy of transferrin as a thermosensitizer in a local 13.56 MHz radiofrequency hyperthermia. (**a**) The *in vivo* evaluation results of oncotherapeutic efficacy of transferrin as a thermosensitizer in the local 13.56 MHz radiofrequency hyperthermia in a tumor-xenografted mouse model. Tumor-xenografted mice received intravenous injections of apotransferrin or transferrin followed by local 13.56 MHz radiofrequency hyperthermia every three days for 5 weeks, and efficacy was evaluated weekly. (**b**) The representative *in vivo* bioimage of the tumor-xenografted mice to evaluate the oncotherapeutic efficacy of transferrin as a thermosensitizer in 13.56 MHz radiofrequency hyperthermia. (**c**) The representative histological examination results of the cancer tissues of the tumor-xenografted mice after each different treatment. The abbreviation in this figure, Ctrl: control mice with apotransferrin injection, Ctrl + hyperthem.: control mice with apotransferrin injection followed by local treatment of 13.56 MHz radiofrequency hyperthermia, transferrin; mice with transferrin injection only, transferrin + hypertherm.: mice with transferrin injection as a thermosensitizer followed by local treatment of 13.56 MHz radiofrequency hyperthermia, paclitaxel: mice in which equivalent dose of paclitaxel was injected as a chemotherapeutic agent without radiofrequency hyperthermia treatment. The values represent the mean ± standard deviation (*n* = 6). A paired Student’s t-test was used for the statistical analysis; ***p < 0.001. Scale bar, 50 µm.

A local 13.56 MHz radiofrequency hyperthermic treatment was repeated every 3 days after *i*.*v*. injection of 10 mg/kg/d transferrin onto the cancer-bearing mice. The local radiofrequency hyperthermia combined with transferrin as a thermosensitizer dramatically reduced the size of cancer tissue as the treatment continued and completely eradicated cancer after 5 weeks of treatment (Fig. 6a,b). The overall anticancer efficacy of the radiofrequency hyperthermia using transferrin as a thermosensitizer was much higher than the oncotherapeutic efficacy by paclitaxel (Fig. 6a,b). In accordance to the dramatic efficacy, H&E staining revealed a typical display of massive cell death by necrosis on the tumor tissues (Fig. 6c). The typical necrosis features, such as formation of swollen cytoplasm, early loss of membrane integrity, and disintegration (swelling) of organelles, became more obvious in the cancer cells of the transferrin-treated group as treatments were repeated (Fig. 6c). Although massive necrosis was obvious everywhere in the transferrin-treated group, necrosis was not observed in any other experimental groups (Fig. 6c). These results indicate that the radiofrequency hyperthermal treatment with transferrin successfully triggers massive necrosis of cancer tissue, suggesting that using transferrin as a thermosensitizer can dramatically increase the oncotherapeutic efficiency of current radiofrequency hyperthermia.

## Discussion

Intrinsic limitation of current cancer therapies prompted us to develop and apply various new technologies for treating cancer. Radiofrequency hyperthermia for cancer is an example of our constant attempt to develop safer and more effective cancer treatment. Application of radiofrequency hyperthermia for treating cancer has been in use for several decades^24^, but ironically, the main obstacle for this treatment stems from limitation to temperature elevation in cancer tissues because it is unable to selectively elevate temperature of cancer tissues. In order to prevent the surrounding normal tissues from being damaged, current radiofrequency hyperthermia generally aims to elevate the temperature of cancer tissues to the range of 40~42 °C. However, temperature elevation to 40~42 °C cannot kill cancer cells efficiently, thereby resulting in death of only a few cancer cells through apoptosis in current radiofrequency hyperthermia^25^. Considering this, it would be hard to expect that current radiofrequency hyperthermia can affect all the area of cancer tissues to eradicate cancer. Thus, the limitation on temperature explains slight oncotherapeutic efficacy of current radiofrequency hyperthermia.

Transferrin is a blood plasma glycoprotein that is encoded by the *TF* gene^26^. A transferrin loaded with ferric ion binds with a transferrin receptor on the surface of a cell to be transported into the cell by receptor-mediated endocytosis^27^. After delivering ferric ion into the cell, the receptor bound with transferrin is out-transported back to the cell surface through exocytosis, releasing apotransferrin for another round of iron uptake^28^. Apotransferrin is present as a much more dominant form than transferrin in blood plasma, which constitutes one of the main serum components^29^. Because transferrin is a naturally abundant serum protein, transferrin is a safe material for medicinal applications. This work showed that transferrin led to selective accumulation of ferric ion in cancer cells. The accumulated ferric ion acted as a thermosensitizer to dramatically boost dielectric heating in a radiofrequency hyperthermia. Considering these characteristics, we believe that transferrin is the ideal choice as a thermosensitizer for treating cancer using radiofrequency hyperthermia.

Application of current radiofrequency hyperthermia for cancer results only few cancer cells to die through apoptosis^30^. In contrast, when combined with transferrin injection, radiofrequency hyperthermia resulted in massive necrosis in the cancer tissues (Fig. 6c). Necrosis is a premature death of cells by autolysis, which is caused by infection, toxins, trauma or high heat^31^. Unlike apoptosis, which is triggered by gentle external factor, necrosis is triggered by harsh external factors such as trauma or high heat^32^. Current radiofrequency hyperthermia lets the temperature of cancer cells to increase to the range of 40~42 °C^6^, which rarely harms cells, only allowing a few cells to die through apoptosis^33^. It would be unrealistic to expect that such a slight elevation in temperature could treat cancer efficiently. This work shows that application of transferrin as a thermosensitizer in radiofrequency hyperthermia successfully increased temperature selectively in cancer tissues, allowing the temperature of cancer tissues to increase up to 47 °C while minimally affecting normal tissues. This method is much more effective oncotherapeutically since selective elevation of temperature allows the temperature to rise high enough to cause necrosis of cancer cells without damaging normal tissues so as to eradicate cancer. Because current radiofrequency hyperthermia are slightly effective in cancer treatment, development of transferrin as a thermosensitizer in radiofrequency hyperthermia would be a major progress in cancer treatment.

Electromagnetic waves such as X-ray, red light, microwave and radiofrequency wave are widely used in modern medicine for various therapeutic purposes (Table 2). Although use of X-ray and red light is limited to cancer treatment^34–39^, microwave and radiofrequency have been broadly used for therapeutic purposes in modern medicine as in the cases of cancer treatment^40^, infectious disease treatment^41^, spinal cord repair^42^, enlarged prostate treatment^43–45^, *etc*^46^. However, unlike X-ray and red light, a sensitizing agent for microwave and radiofrequency has not been developed. It is our opinion that transferrin or related agents could be broadly used as sensitizing agents for oncotherapeutics using microwave and radiofrequency in near future.

**Table 2:** Electromagnetic waves widely used in modern medicine.

Electromagnetic wave	Wavelength	Sensitizing agent	Clinical Use	Study
X-ray	0.01~10 nm	Misonidazole, metronidazole, tirapazamine, fluoropyrimidines, gemcitabine, cisplatin, *etc*	Cancer	Sheldon, *et al*.^34^, Raviraj, *et al*.^35^, Rousseau, *et al*.^36^
Red light	600~700 nm	porphyrins, chlorophylls, dyes, aminolevulinic acid, *etc*	Cancer, age-related macular degeneration, psoriasis, atherosclerosis	Vicente, *et al*.^37^, Yoon, *et al*.^38^, Ormond, *et al*.^39^
Microwave	1 × 10^6^~1 mm	None	Cancer	Rubeinstein, *et al*.^40^
Radiofrequency	1 mm~100 km	None	Cancer, infectious disease, spinal cord injury, enlarged prostate, *etc*	Sadeghian *et al*.^41^, Akkoc, *et al*.^42^, Zlotta, *et al*.^43^, Yerushalmi, *et al*.^44^, Sapozink, *et al*.^45^, Habash, *et al*.^46^

The clinical applications of electromagnetic wave in modern medicine are summarized from previously published data^34–46^.

## Methods

### Culture of cancer cell

The lung human cancer cell line, NCI-H460-luc2 (Caliper Life Sciences) was used to assess the efficacy of transferrin as a thermosensitizer in radiofrequency hyperthermia. The cancer cell line was cultured in RPMI-1640 medium (HyClone) supplemented with 10% heat-inactivated fetal bovine serum (HyClone) and 1% antibiotics (100 U/mL penicillin and 0.1 mg/mL streptomycin, HyClone) at 37 °C in a humidified incubator containing 5% CO_2_.

### Isolation and culture of normal cells

Adipose tissue-derived mesenchymal stem cells (AT-MSCs) used as a normal cell control were obtained from the peritoneal adipose tissue excised from the abdominal region of C57BL/6 male mouse. The adipose tissue was first cut into fine pieces and enzymatically digested with 0.2% collagenase (Sigma Aldrich) in PBS (Sigma Aldrich) for 1 hr at 37 °C with gentle agitation to isolate AT-MSCs. After isolation of AT-MSCs, the collagenase was inactivated by adding an equal volume of DMEM (HyClone) supplemented with 10% FBS and centrifuged at 400 × g for 5 minutes at room temperature. The resulting cell pellet was suspended in 0.83% NH_4_Cl (Sigma Aldrich), incubated for 2 min to eliminate red blood cells and filtered through a 100 µm mesh (BD Biosciences) to remove cell aggregates and connective tissue debris. The cell pellet was then collected by centrifugation at 400 × g for 5 minutes, resuspended in a Mesencult^TM^ medium (STEMCELL) supplemented with mesenchymal stem cell stimulatory supplements (STEMCELL), and plated in a collagen-coated T175 culture flask (BD Biosciences). The isolated AT-MSCs were maintained at 37 °C in a 5% CO_2_ incubator. After 12∼16 hours, the non-adherent dead AT-MSCs were removed by changing the medium, and adherent AT-MSCs were cultured for further expansion. At 70~80% confluence, they were trypsinized and sub-cultured at a density of 5 × 10^3^ cells/cm^2^ in a T175 culture flask for further experiments.

### *In vitro* thermal sensitivity test of transferrin in radiofrequency wave

To evaluate the thermal sensitivity of transferrin (Sigma Aldrich) and apotransferrin (Sigma Aldrich) under radiofrequency wave, 0.1 mL of the 0, 0.04, 0.2, 1, or 5 mg/mL solutions was seeded into 96-well plates three times per sample. After seeding, the 13.56 MHz radiofrequency wave (Oncotherm) at 50 W energy dose was exposed to samples for 10 minutes by using a radiofrequency hyperthermal equipment designed for human patient (Oncotherm). The temperature elevations of each solution were measured by the thermal imaging camera (FLIR), and the temperatures of each solution was analyzed by DirA Program (FLIR Tools).

### *In vitro* evaluation of transferrin as a thermosensitizer in radiofrequency hyperthermia

The NCI-H460 cells and AT-MSCs were used to assess the boosting effect of temperature elevation by transferrin *in vitro* as a thermosensitizer in 13.56 MHz radiofrequency hyperthermia. The NCI-H460 cells (1 × 10^3^ cells/mL) and AT-MSCs (3 × 10^3^ cells/mL) were first seeded into 96-well plates, followed by maintaining at 37 °C in a humidified incubator containing 5% CO_2_ for 12 hours. After stabilizing the seeded cells in 96-well plates, the NCI-H460 cells and AT-MSCs were treated with transferrin at different concentrations (0, 0.04, 0.2, 1, 5 mg/mL) and incubated for 4 hours. The cells were then washed twice with culture media, followed by adding distilled water. The cells in distilled water were exposed to 13.56 MHz radiofrequency wave for 10 minutes at 50 W energy dose by using the radiofrequency hyperthermal equipment designed for human patient (Oncotherm). The boosting effect of temperature elevation by transferrin as a thermosensitizer in radiofrequency hyperthermia was measured by the thermal imaging camera (FLIR), and the temperature of the solutions was analyzed by DirA Program (FLIR Tools). See the supplementary information for detailed methods.

### Animal studies

This study was carried out in strict accordance with the recommendations in the Guide for the Ethics Committee of Chonbuk National University Laboratory Animal Center. The protocol was approved by the Ethics Committee of Chonbuk National University Laboratory Animal Center (Permit Number: CBU 2012–0040). All efforts were made to minimize suffering. Female nude mice with a BALB/c genetic background were purchased from Damool Sicences Co. at 6 weeks of age, and housed 5 in each cages with food and water available *ad libitum* unless otherwise stated. They were maintained under a 12 hr/12 hr light/dark cycle at a temperature of 22 °C and humidity of 55 ± 5%. The Animal care protocol was followed as previously described^47–49^. After stabilizing the nude mice in the cages, The NCI-H460 (5 × 10^6^) cells were injected subcutaneously into the nude mice weighting 21 to 25 g. The growth of tumor in the tumor-bearing mice was monitored twice weekly by measuring the longest (L) and shortest (W) tumor diameters (mm) using caliper. The formula for an ellipsoid sphere [(L × W^2^)/2] was used to calculate the tumor volume. The volume was converted to tumor weight assuming unit density (i.e., 1 mm^3^ = 1 mg). When the tumors reached a size of ~100 mg, the tumor-bearing mice were used for *in vivo* assessment of transferrin as a thermosensitizer in radiofrequency hyperthermia.

### *In vivo* evaluation of transferrin as a thermosensitizer in radiofrequency hyperthermia

When the tumors reached a size of ~100 mg, the mice (*n* = 3) were *i*.*v*. injected with transferrin solution (10 mg/kg/d). The transferrin solution was *i*.*v*. injected every 3 days into the tumor-bearing mice. The tumor tissues of the tumor-bearing mice were locally exposed to 13.56 MHz radiofrequency wave 4 hours later after injection of transferrin by using a radiofrequency hyperthermal equipment designed for rodents (Oncotherm) for 1 minute at 2 W energy dose. The effect of transferrin in a whole-body radiofrequency hyperthermia was assessed by irradiating 13.56 MHz radiofrequency wave onto the tumor-bearing mice for 30 minutes at 100 W energy dose 4 hours later after injection of transferrin by using a radiofrequency hyperthermal equipment designed for human patient (Oncotherm). The boosting effect of temperature elevation by transferrin in both local and whole-body exposure to13.56 MHz radiofrequency wave was measured by a thermal imaging camera (FLIR). The detailed temperature measurement method on cancer and normal subcutaneous areas is described supplementary information.

### Distribution of ferric ion in tumor-bearing mice

A small pieces (~1 g) of each organ including cancer tissue were isolated from the tumor-bearing mice *i*.*v*. injected with transferrin at different time point. After measuring the weight of the pieces of the mouse organs, the organ pieces were dried for 24 hours in hood. Two mL of 6N HCl was added into each of the completely dried organ pieces in hood, and the glass bottles were incubated in water-bath at 55 °C for 24 hours to solubilize the organ pieces. After incubation, the solubilized organs were transferred to 15 mL centrifuge tubes (SPL) for centrifugation at 1,000 rpm for 15 minutes, and each of the supernatants were collected for quantitation of ferric ion. The ferric ion concentrations of each organ were measured by a inductively coupled plasma mass spectrometry (Varian).

### Evaluation of oncotherapeutic efficacy of transferrin as a thermosensitizer in a 13.56 MHz radiofrequency hyperthermia

When xenografted tumors reached a size of ~100 mg (~10 days after inoculation), mice (*n* = 6) subsequently received intravenous injections of transferrin solution (10 mg/kg/d) every three days for 5weeks. After 4 hours of *i*.*v*. injection of transferrin or apotransferrin, 13.56 MHz radiofrequency wave was irradiated onto the tumor tissue for 1 min at 2 W energy dose by using a local radiofrequency hyperthermal equipment designed for rodents (Oncotherm). For comparison of oncotherapeutic efficacy of transferrin as a thermosensitizer in the radiofrequency hyperthermia with conventional chemotherapy, tumor-bearing mice (*n* = 6) received intravenous injections of cremophor-based paclitaxel (10 mg/kg/d, Bristol-Myers Squibb SRL) every three days for 5 weeks. The oncotherapeutic efficacy was assessed by measuring *in vivo* imaging on days 7, 14, 21, 28 and 35 at weekly intervals. A D-luciferin firefly potassium salt (15 mg/mL, PerkinElmer) in PBS was filtered through 0.2 μm filters (Merck Millipore) before use. Mice were injected intraperitoneally with D-luciferin firefly potassium salt (150 mg luciferin/kg body weight). The mice were exposed for 1 min, beginning 12 minutes after the injection of D-luciferin, to generate a bioluminescent image using an IVIS imaging system (PerkinElmer). Bioluminescence color images were superimposed using the living image software. Data were analyzed with Igor Pro imaging analysis software (WaveMetrics). A region of interest (ROI) was manually selected over signal intensity. The area of the ROI was kept constant. Data are presented as average radiance (photon/sec) within the ROI.

### Histology of tumor tissue

The Histological analysis protocol was followed as previously described^49^. The tumor tissue specimens from the cancer-bearing mice of experimental groups were routinely isolated. After tumor tissues were fixed at 10% neutral-buffered formalin (Sigma Aldrich), the samples were processed for staining with Hematoxyline and Eosin (H&E) as previously described^49^. The stained tissue images were obtained by Aperio Scanscope FL (Leica Biosystems) and processed by ImageScope Software (Aperio Technologies).

### Statistics

All data are presented as the mean ± standard deviation and were compared using paired Student’s t-tests. *P* values < 0.05 was considered as the statistical significance level.

## Electronic supplementary material

Supplementary Information
