# Supplementary material for: Transferrin as a thermosensitizer in radiofrequency hyperthermia for cancer treatment
Source: Sci Rep. 2018 Sep 10;8:13505. doi: 10.1038/s41598-018-31232-9 (PMC6131143; doi:10.1038/s41598-018-31232-9)
Supplement: Supplementary file 1 — Supplementary Information [file 41598_2018_31232_MOESM1_ESM.docx]

**Supplementary Information**

**Transferrin as a thermosensitizer in radiofrequency hyperthermia for cancer treatment**

Hea-Jong Chung, Heui-Kwan Lee, Ki Beom Kwon, Hyeon-Jin Kim & Seong-Tshool Hong^*^

**Contents:**

Supplementary methods

Supplementary Figure 1, 2, 3, 4, 5, 6, 7, 8, 9

**Supplementary methods**

**Measurement of temperature by DirA program after thermal photography.** The boosting effect of temperature elevation by transferrin as a thermosensitizer in radiofrequency hyperthermia was measured by FLIR thermal imaging camera, and the thermal images was analyzed by DirA Program (FLIR Tools) to measure the temperature. The mice were photographed with FLIR thermal imaging camera. The thermal images were analyzed by DirA Program (FLIR Tools) to measure the temperature. Because the DirA Program can spot every pixel to measure temperature accurately, 25 spots of on each of the cancer and normal subcutaneous areas randomly allocated on thermal images. The digital values of temperature measurements on each spot were averaged to determine the temperature of the cancer and normal subcutaneous areas of the mice. The temperature was measured before and after 13.56 MHz radiofrequency hyperthermia for temperature change (ΔT). All data are presented as the mean ± standard deviation and were compared using paired Student’s t-tests. *P* values < 0.05 was considered as the statistical significance level.


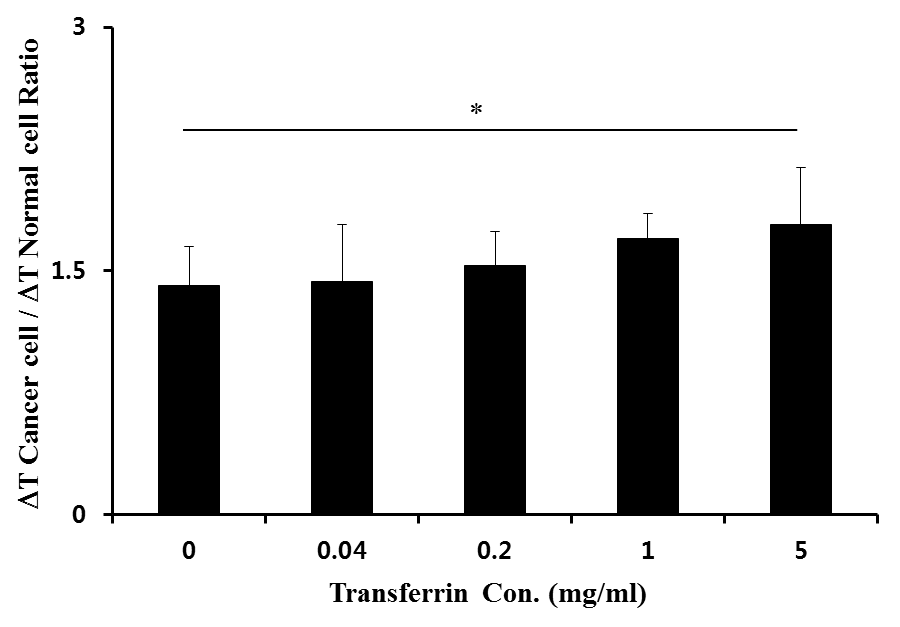


**Supplementary Fig. 1** The effect of transferrin exposure to cancer and normal primary cells. Exposure to transferrin induced dielectric heat more effectively in cancer cells than in normal primary cell in the 13.56 MHz radiofrequency wave. The temperature change after the exposure to the 13.56 MHz radiofrequency wave in each experiment was measured by FLIR thermal camera, and the temperature change of cancer cells was divided by those of normal cells. The values represent the mean ± standard deviation (*n* = 6). A paired Student’s t-test was used for the statistical analysis; * *p* < 0.05.


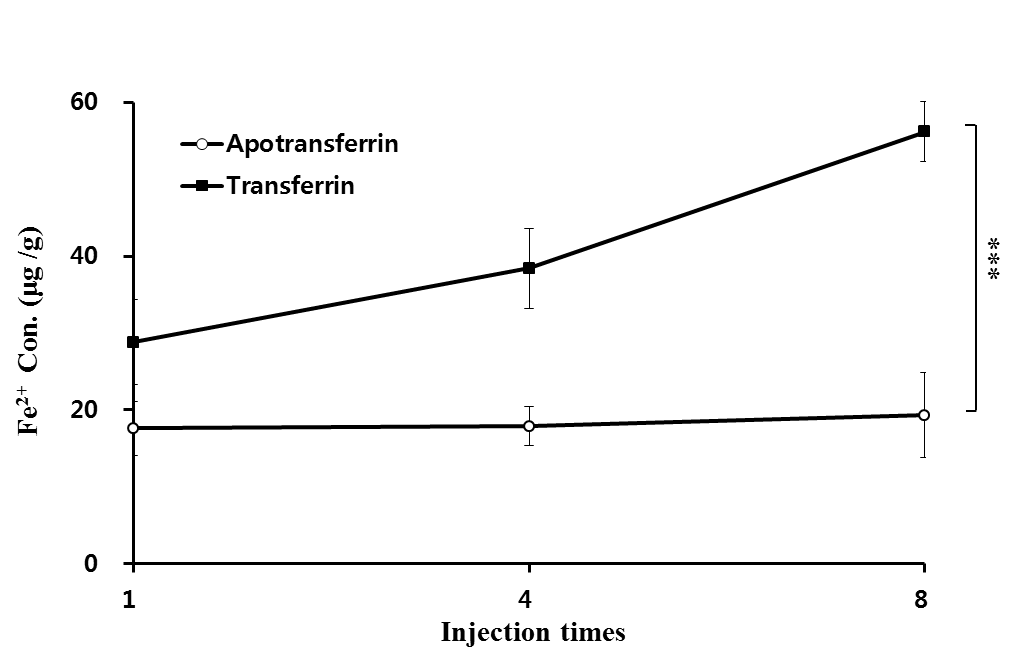


**Supplementary Fig. 2** The effect of repeated *i.v.* injection of transferrin into tumor-bearing mice. Quantity of ferric ion in the cancer tissues of the tumor-bearing mice was increased by repeated *i.v.* injection of transferrin. The ferric ion in the tumor tissues of the tumor-bearing mice was quantitated by an ICP-MS method after complete acid lysis of the tumor tissues. The values represent the mean ± standard deviation. (*n* = 6). A paired Student’s t-test was used for the statistical analysis; * * * *p* < 0.001.


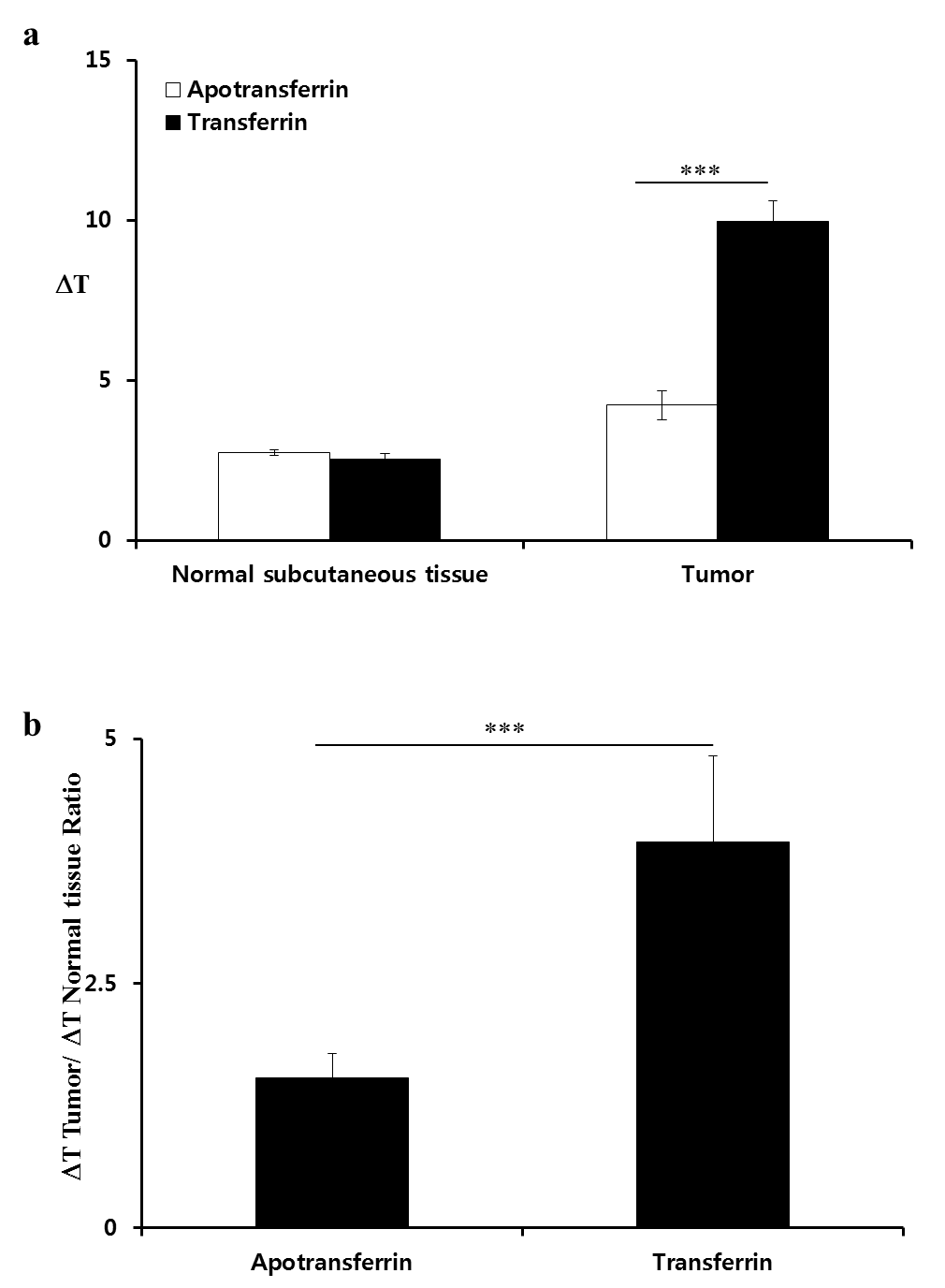
**Supplementary** **Fig. 3** Application of transferrin as a thermosensitizer in a local 13.56 MHz radiofrequency hyperthermia boosted cancer-selective temperature elevation in tumor-bearing animals. The temperature elevation after the local 13.56 MHz radiofrequency hyperthermia was measured by FLIR thermal camera after single injection of apotransferrin or transferrin. The temperature was measured before and after the local radiofrequency hyperthermia. (**a**) Injection of transferrin led to a selective temperature elevation in cancer tissue by the local 13.56 MHz radiofrequency hyperthermia. (**b**) The temperature change of cancer tissues was divided by those of normal subcutaneous tissue to show the effect of transferrin as a thermosensitizer. The values represent the mean ± standard deviation (*n* = 6). A paired Student’s t-test was used for the statistical analysis; * * * *p* < 0.001.


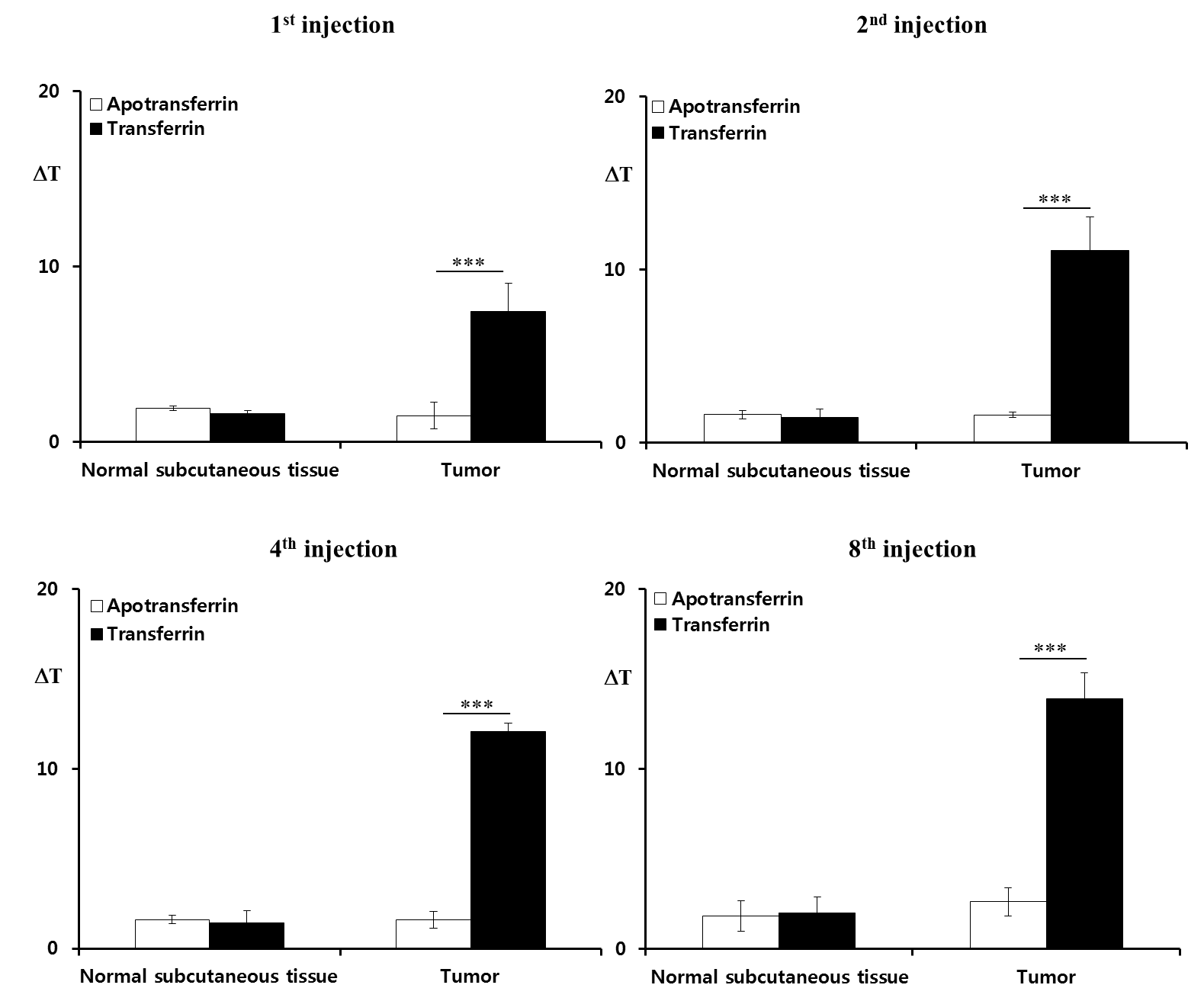


**Supplementary Fig. 4** The positive co-relationship between the cancer-selective temperature elevation and ferric ion accumulation in a local 13.56 MHz radiofrequency hyperthermia. As ferric ion accumulated in cancer cells by repeated *i.v.* injection of transferrin (see Supplementary Fig. 2), the cancer tissues became more sensitive to the local 13.56 MHz radiofrequency hyperthermia, resulting in boosted temperature elevations. The values represent the mean ± standard deviation (*n* = 6). A paired Student’s t-test was used for the statistical analysis; *** *p* < 0.001.


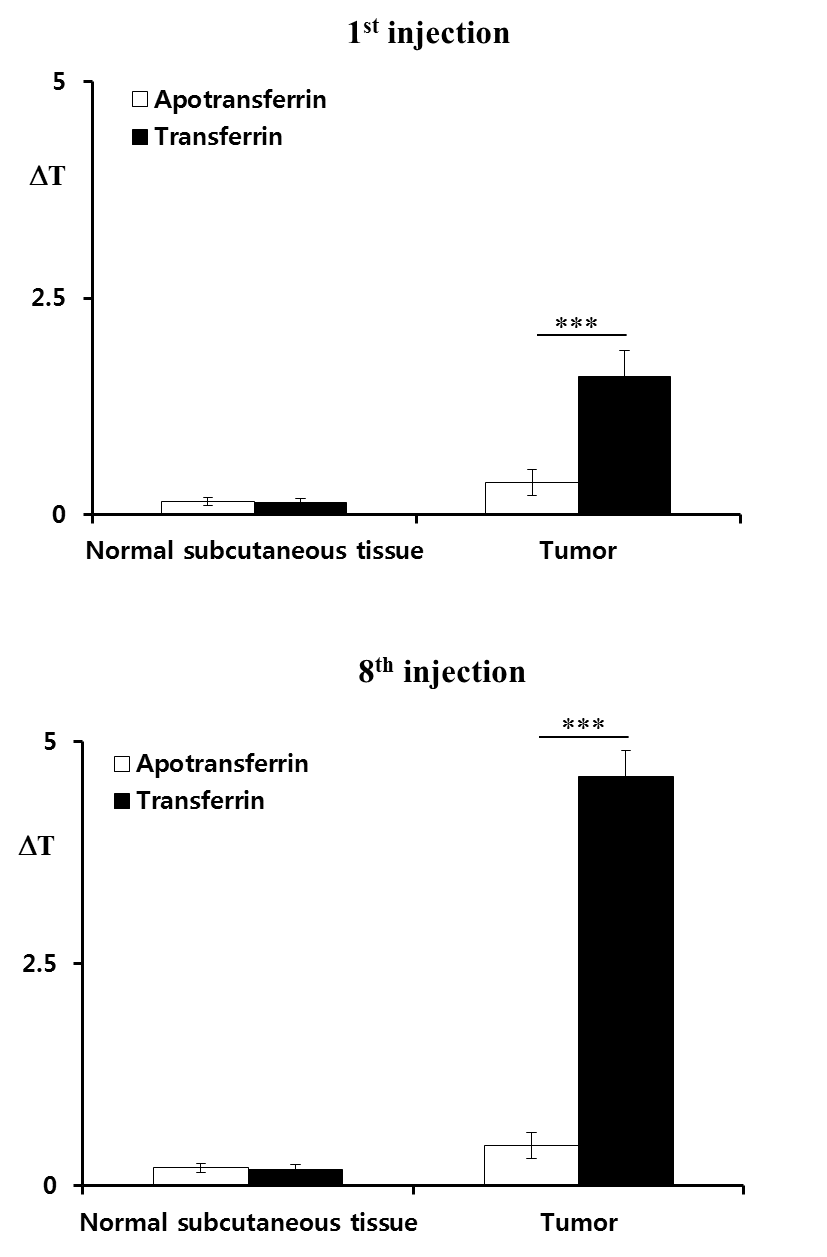


**Supplementary Fig. 5** The positive correlation between the cancer-selective temperature elevation and ferric ion accumulation in a whole-body 13.56 MHz radiofrequency hyperthermia. As ferric ion accumulated in cancer cells by repeated *i.v.* injection of transferrin (see Supplementary Fig. 2), the cancer tissues became more sensitive to the whole-body 13.56 MHz radiofrequency hyperthermia, resulting in boosted temperature elevations. The values represent the mean ± standard deviation (*n* = 6). A paired Student’s t-test was used for the statistical analysis; *** *p* < 0.001.


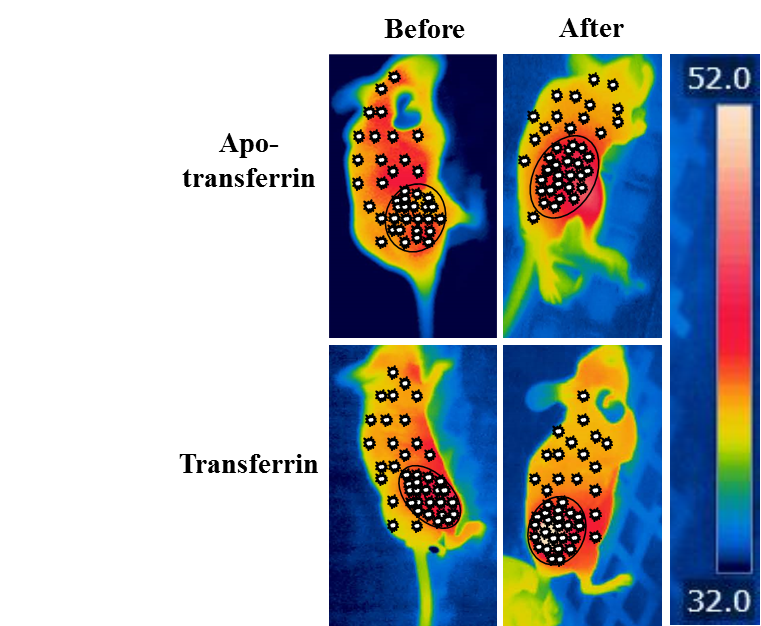


**Supplementary Fig. 6** The Thermal images showing how the temperatures of cancer and normal subcutaneous areas were determined in Figure 2. After identifying cancer area on the thermal images by circling to distinguish the cancer areas and normal subcutaneous areas, 25 spots of on each of the cancer and normal subcutaneous areas randomly allocated. The digital values of temperature measurements on each spot were averaged to determine the temperature of the cancer and normal subcutaneous areas of the mice.

**
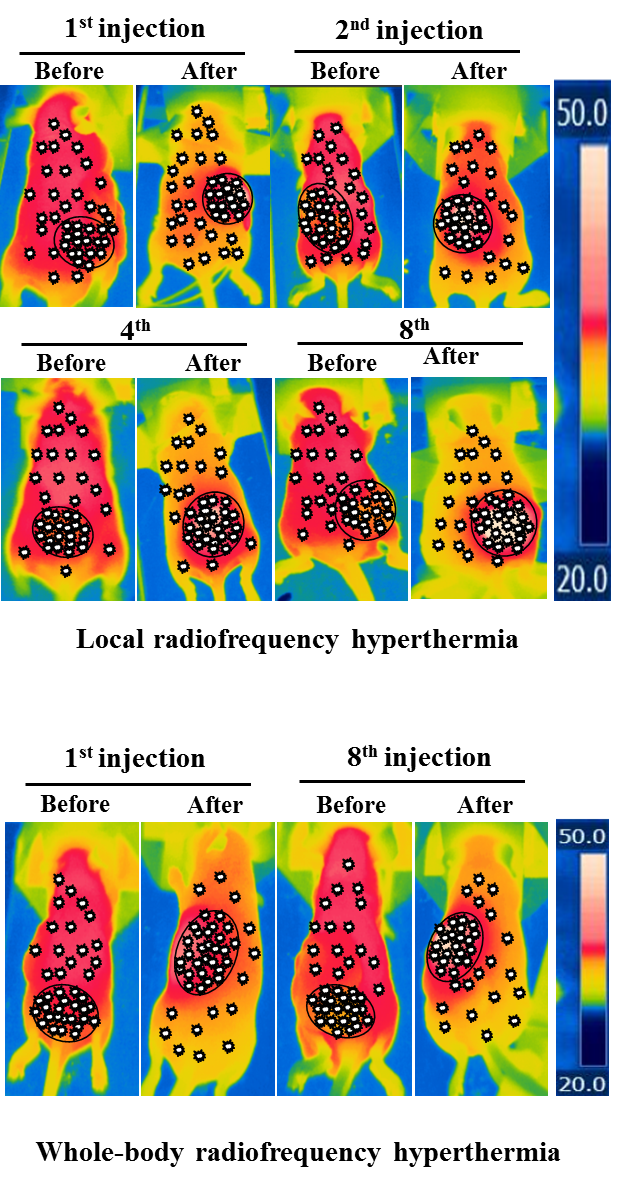
**

**Supplementary Fig. 7** The Thermal images showing how the temperatures of cancer and normal subcutaneous areas were determined in Figure 3. After identifying cancer area on the thermal images by circling to distinguish the cancer areas and normal subcutaneous areas, 25 spots of on each of the cancer and normal subcutaneous areas randomly allocated. The digital values of temperature measurements on each spot were averaged to determine the temperature of the cancer and normal subcutaneous areas of the mice.

**
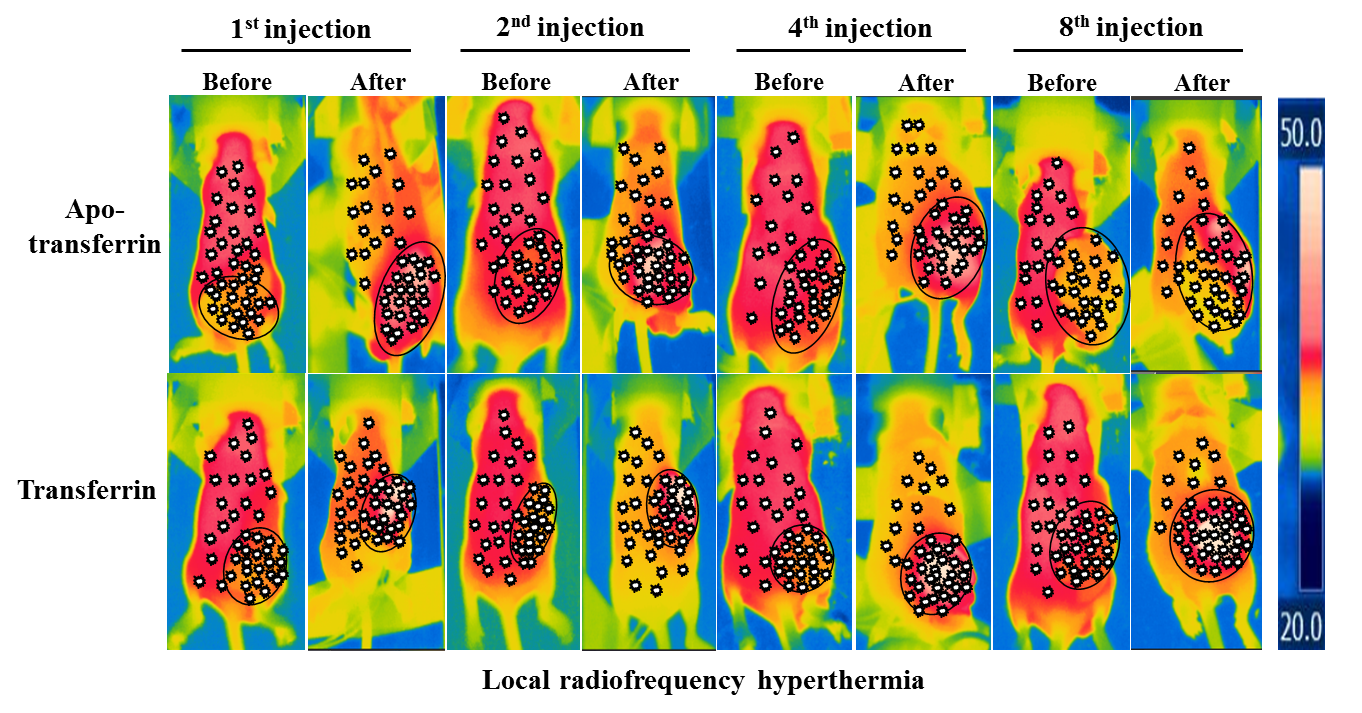
**

**Supplementary Fig. 8** The Thermal images showing how the temperatures of cancer and normal subcutaneous areas were determined in Figure 4. After identifying cancer area on the thermal images by circling to distinguish the cancer areas and normal subcutaneous areas, 25 spots of on each of the cancer and normal subcutaneous areas randomly allocated. The digital values of temperature measurements on each spot were averaged to determine the temperature of the cancer and normal subcutaneous areas of the mice.


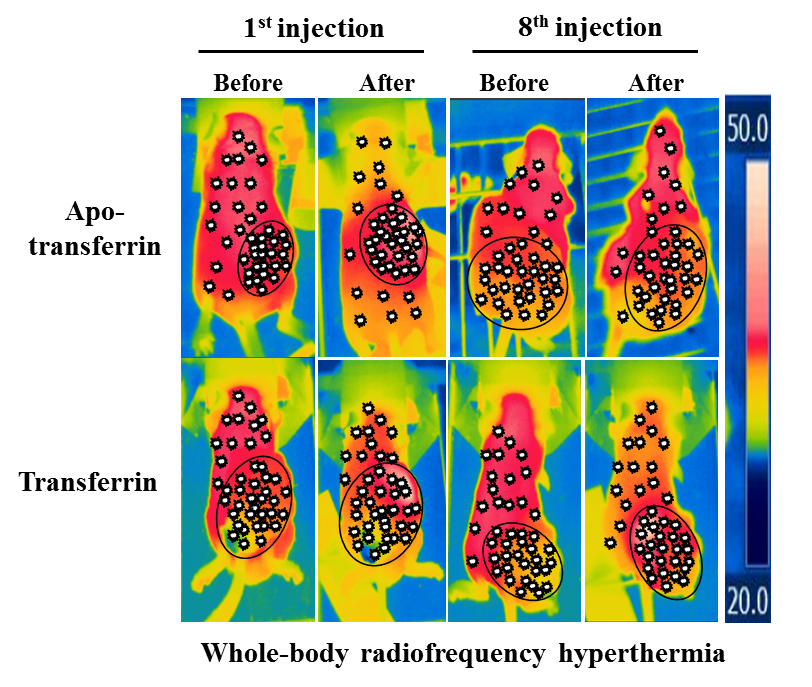


**Supplementary Fig. 9** The Thermal images showing how the temperatures of cancer and normal subcutaneous areas were determined in Figure 5. After identifying cancer area on the thermal images by circling to distinguish the cancer areas and normal subcutaneous areas, 25 spots of on each of the cancer and normal subcutaneous areas randomly allocated. The digital values of temperature measurements on each spot were averaged to determine the temperature of the cancer and normal subcutaneous areas of the mice.
